# Supplementary material for: Are carbon emissions trading and green financial instruments synergistic? -Comprehensive quantitative research based on content analysis
Source: PLoS One. 2024 Mar 7;19(3):e0298601. doi: 10.1371/journal.pone.0298601 (PMC10919601; doi:10.1371/journal.pone.0298601)
Supplement: S1 Data — (ZIP) [file pone.0298601.s001.zip › Data/地级市Z_F工作报告.zip!/║╙─╧/┐¬╖Γ╩╨/└┤╫╘╣1⁄2╓┌║┼í╛┬φ┐╦╩2╛▌═°í┐_2015.docx]

各位代表：

　　现在，我代表市人民政府，向大会报告工作，请予审议，并请各位政协委员和其他列席人士提出意见。

　　一、2014年工作回顾

　　2014年是全市人民备受鼓舞的一年，习近平总书记亲临开封视察指导，充分体现了党中央对开封的亲切关怀。2014年是我市经济社会各项事业取得新成绩的一年，全市上下在省委、省政府和市委的正确领导下，在市人大、市政协的监督支持下，认真学习贯彻习近平总书记系列重要讲话精神，坚持“好中求快”总基调，全力打好“五大攻坚战”，务实重干，奋力拼搏，较好完成了市十四届人大一次会议确定的各项任务。

　　——经济实力不断增强。全市生产总值完成1492.1亿元，比上年增长9.5%，其中第一产业增加值285.2亿元，增长4.4%；第二产业增加值678亿元，增长11.5%；第三产业增加值528.9亿元，增长9.7%。一般公共预算收入96.2亿元，增长19.1%。固定资产投资1135.6亿元，增长20.6%。社会消费品零售总额653.1亿元，增长13%。城镇居民人均可支配收入21467元，农村居民人均纯收入9316元，分别增长10.1%和11.5%。

　　——发展速度持续领先。生产总值增幅居全省第四位，连续四年超过全省平均水平。规模以上工业增加值、一般公共预算收入、固定资产投资、社会消费品零售总额增幅分别位居全省第一位、第二位、第二位和第三位，主要经济指标增幅继续位居全省前列。

　　——发展质量明显提升。产业结构更加优化，三次产业结构达到19.1∶45.4∶35.5，一产比重首次降至20%以下，二三产比重比上年提高1.5个百分点。税收占一般公共预算收入比重达到71.8%。经济发展内生动力进一步增强，全市358个重点项目完成投资858亿元，是近年来投资规模最大的一年。

　　——城乡面貌显著改观。城乡一体化示范区建设加快推进，老城区改造力度不断加大，清洁家园·美丽乡村活动和小城镇综合整治提升工程成效明显。众志成城，成功创建国家卫生城市、省级文明城市和省级旅游标准化示范城市，十大“开封规矩”初步形成。

　　——民生进一步改善。在财政支出压力增大的情况下，投入民生资金156.2亿元，占一般公共预算支出的70%。十项民生工程全面完成，一大批群众关切的热点难点问题得到解决。

　　——社会大局和谐稳定。信访工作进入全省前列，全市没有发生重大安全生产和食品药品安全事故，平安开封建设取得新成效，实力开封、文化开封、美丽开封、幸福开封建设迈出新步伐。

　　一年来，我们主要做了以下工作：

　　（一）打好工业强市攻坚战，工业经济平稳运行。全市规模以上工业企业达到1293家，增加39家；规模以上工业增加值完成519.7亿元，增长13.7%。工业用电量增长20.3%，增幅居全省第一位。50家重点培育企业和50家高成长型企业稳定发展，晋开集团产值超60亿元，奇瑞汽车和瑞创农机产值超30亿元，中电投开封发电公司、兴化精细化工公司实现利润分别超4亿元和1亿元，平原非标在新三板挂牌上市。十大产业集群实现主营业务收入2029.7亿元，增长11.6%。强力推进工业项目建设，134个重点工业项目完成投资418亿元，增长21.3%，正隆包装、沃隆车业等60个工业项目建成投产。加快产业集聚区发展，8个产业集聚区完成固定资产投资729.4亿元，增长19%，其中基础设施投资完成119.1亿元，增长15.8%。通许产业集聚区被评为“省十快产业集聚区”，汴西产业集聚区被评为“二星级产业集聚区”，尉氏和杞县产业集聚区被评为“一星级产业集聚区”。

　　（二）加快城乡一体化示范区建设，新区形象快速提升。多元化融资70.3亿元，完成基础设施投资27.4亿元。开封西湖蓄水成景，郑开城际铁路开通运营，郑开大桥、东京大桥建成投用，陇海四路、陇海五路、十八大街、十九大街等道路建成通车，郑开大道拓宽、金明大道北段改造等一批道路管网工程有序推进。中心商务区、核心区内79个重点项目完成投资128.7亿元，“八园两馆一中心”等项目进展顺利。着力培育主导产业，新引进亿元以上项目36个，中南建材城、奇瑞微车、大商麦凯乐购物中心、银基水上大世界等一批重大项目成功落户，德豪光电、武秀汽车配件、泰丰生物制药等一批主导产业项目快速推进。

　　（三）加强文商旅融合，第三产业健康发展。成功举办2014中国（开封）清明文化节、世界客属第27届恳亲大会、中国开封第32届菊花文化节、中国收藏文化（开封）论坛、第八届中国郑开国际马拉松赛。“一河两街三秀”品牌效应进一步扩大，小宋城、七盛角民俗文化街区建成开业，守望阁、珠玑巷、城墙保护五期工程建成开放，东京极地海洋馆正式运营，中原明珠文化产业园、双龙巷历史文化街区、汴京公园及周边环境改造等重点文化旅游项目扎实推进。全市旅游接待量、旅游综合收入分别增长13.6%和15.9%。

　　金融业平稳发展，人民币各项存款余额1274.1亿元，同比增长12.3%；人民币各项贷款余额864.4亿元，同比增长23.6%，增幅位居全省第二。新增存贷比上升到118%。全市金融机构实现税收8.2亿元，增长42.1%。成功发行第二期城投债。实现直接融资112.8亿元、间接融资165亿元。商贸、邮政、电信、交通运输、房地产业健康发展，第三产业增加值占生产总值的比重连续五年位居全省前列。

　　（四）积极推进新型城镇化，城乡建设步伐加快。新一轮行政区划调整基本完成，设立祥符区，市区面积由547平方公里扩大到1849平方公里，市区人口由91万增加到169万，拓展了城市发展空间，预计全市城镇化率达到43%。强力开展“两改一建”工程，完成棚户区、城中村改造281.4万平方米。不断完善城市基础设施，启动城区“三贯通”工程和火车站周边开发改造，基本完成内顺城路（向阳路—西门大街）改造、文庙街改造、西环路北延（东京大道—复兴大道）等基础设施建设工程，内环路改造、华夏大道改造、官坊立交辅道及排水工程、东京大道雨水干渠工程等基础设施建设步伐加快。郑徐客运专线、郑民高速二期、新开铁路、商登高速、机西高速等项目开封段扎实推进。实施规划提升、以产兴城等县城建设十大工程，县城和中心镇面貌明显改观。加强农村基础设施建设，新改建农村公路282公里，改造农村危房1.2万户，解决33.4万农村居民和在校师生安全饮水问题，农村生产生活条件进一步改善。

　　（五）大力发展农业产业化，农业基础不断巩固。推进高标准粮田“百千万”工程建设，粮食总产达278.6万吨，增长2.2%，实现“十一连增”。农业产业化进程不断加快，现代农业产业化集群达到8个，省级农业产业化龙头企业增加到26家。积极培育新型农业经营主体，农民合作社发展到6620家，家庭农场达到490家，累计流转土地140.9万亩。农业机械化率达到81%。茂盛粮机工程技术研究中心通过国家验收。全市优质农作物种植面积超过62%，主要农作物良种覆盖率在99%以上。新认定无公害农产品生产基地10个、无公害农产品26个。新增标准化规模养殖场60个，肉蛋奶产量稳定增长。农业基础设施不断完善，柳园口、赵口灌区续建配套与节水改造等工程扎实推进，“油改电”工程顺利实施，新增改善灌溉面积35万亩。

　　（六）扎实推动各项改革，发展动力持续增强。坚持用改革办法解决发展难题，积极推进第一批20项重点改革。市县政府机构改革基本完成。深化审批制度改革，审批事项从318项精简至182项。高标准建成市民之家，群众办事更加方便快捷。加快工商登记制度改革，新登记各类市场主体增长27%。企业改革持续深化，国有资产监管体制进一步完善。推进财税金融体制改革，市区实行新的分税制，“营改增”范围不断扩大。成立开封市民间借贷登记服务公司。村镇银行基本实现县域全覆盖。开展农村产权制度改革试点，基本完成农村集体土地所有权确权登记发证。县级公立医院综合改革正式启动。农电体制基本理顺，户籍制度、医药卫生、水电气价格、文化体制等领域改革取得阶段性成果。

　　（七）加大招商引资力度，对外开放持续扩大。扎实开展对外开放提升年工作和重点产业大招商活动，成功举办“闽粤客商开封行”等招商活动，引进省外项目256个，实际到位省外资金406.4亿元，增长15.2%；实际利用外资5亿美元，增长17.4%；进出口总值4.4亿美元。新批准外商投资企业13家。新培育出口基地3个。坚持引资与引智并重，引进院士、“千人计划”专家、黄河学者20余人。与桂林市缔结友好城市，国内外友好城市达到17个。

　　（八）推动生态文明建设，环境保护不断加强。实施蓝天工程、碧水工程，深化环境综合整治，开展重点领域节能减排低碳行动，全面完成化学需氧量、氨氮、二氧化硫、氮氧化合物减排任务，节能减排完成省下目标。集中整治燃煤锅炉及露天烧烤，加快淘汰黄标车，全市空气优良天数276天，达标率75.6%。实施黄汴河和东护城河截污整治，完成6家污水处理厂一级A提标改造，建成7个重点污水处理工程，6条出境河流断面综合达标率达到73.9%，超额完成省下目标。启动黑池、柳池饮用水源地综合整治，饮用水源水质达标率100%。扎实推进林业生态市建设，新增造林面积10.9万亩。

　　（九）切实为群众谋福祉，民生工程扎实推进。落实国家强农惠农富农政策，发放各类补贴12.6亿元。千方百计扩大就业，全市新增城镇就业6.8万人，动态消除“零就业家庭”，城镇登记失业率控制在3%以内。切实加强社会保障，城镇职工基本养老、基本医疗保险参保人数分别达到62.6万人、97.3万人，城乡居民养老保险参保人数达到182.7万人，企业退休人员基本养老金人均月增加195元。新农合参合人数338.7万人，参合率达99.83%，人均筹资水平提高到380元，住院补偿封顶线提高到20万元。失业、工伤、生育等保险覆盖面继续扩大。社会救助制度全面建立，市社会福利园区投入使用，新增养老服务机构20家、床位1818张。强力推进保障性安居工程建设，开工建设公租房、棚户区改造安置住房12230套，基本建成9040套。持续改善居民生活条件，新增清洁能源1200万立方米，新增集中供热面积221万平方米，新建放心馒头店200家、便民菜店50家，新建升级农贸市场8个，新建改造水冲式厕所842座，改造背街小巷616条。新开通、优化调整公交线路13条，新增公交车100台。建成200个农民体育健身工程、33个乡镇体育工程。完成35个贫困村整村推进建设任务，新实现4万贫困人口脱贫。加大教育卫生投入，开封高中新校区和集英中学建成招生；启动第二期学前教育三年行动计划，新建扩建幼儿园43所；改扩建20个乡镇卫生院，新建55个村卫生室。“单独两孩”生育政策顺利实施，人口自然增长率控制在6‰以内。扎实推进文化惠民工程，认真做好“两馆一站”免费开放工作，组织开展“舞台艺术送农民”活动和公益性电影放映工程。切实做好信访稳定工作，加强安全生产和食品药品安全监管，狠抓社会治安综合治理，严厉打击各类刑事犯罪，社会大局保持和谐稳定，群众安全感和满意度持续上升。

　　一年来，我们自觉接受人大依法监督、政协民主监督和社会监督，认真办理人大代表建议和政协提案1251件，满意率97%；支持民主党派、工商联和无党派人士参政议政。切实加强民主法制工作，认真做好普法宣传教育，大力推进依法行政，全社会法治意识持续增强。不断强化政府自身建设，扎实开展党的群众路线教育实践活动，严格落实中央八项规定，大力弘扬焦裕禄精神，着力解决“四风”问题，加强行政监察和审计监督，持续推进“两转两提”，严格效能问责，行政效能明显提升。加强社会主义核心价值体系学习教育和精神文明创建，公民道德素质和社会文明程度持续提高。加强国防教育、国防后备力量及人民防空建设，做好“双拥”工作，开封驻军和武警部队在支持开封经济建设、维护社会稳定等方面作出新的贡献。民族宗教工作进一步加强。对口援疆工作进展顺利。统计、外事、侨务、港澳、对台、档案、史志、地震、气象、社科研究等工作都取得了新的成绩。

　　各位代表！

　　过去的一年，在宏观环境复杂严峻、经济下行压力加大的情况下，我市经济社会实现了有质量的稳定增长、可持续的全面发展，成绩来之不易。这是省委、省政府和市委正确领导的结果，是市人大、市政协监督支持的结果，是广大干部抢抓机遇、务实重干、团结拼搏的结果，凝聚着全市人民的智慧和汗水。在此，我代表市人民政府，向工作在各条战线上的工人、农民、知识分子、干部，向驻汴解放军指战员、武警官兵和公安民警，向各民主党派、工商联及社会各界人士致以崇高的敬意！向所有关心、支持开封发展的同志们、朋友们表示衷心的感谢！

　　在肯定成绩的同时，我们也清醒地认识到，我市经济社会发展和政府自身建设还存在一些突出问题：综合实力依然偏弱，主要指标总量均在全省中游偏下，底子薄、基础弱、人均水平低的基本市情尚未根本改变。产业结构不合理，一产比重高于全省7.2个百分点，二产比重低于全省5.8个百分点，第三产业发展还不充分，仍低于全国平均水平12.7个百分点。战略谋划不够，科技创新能力不强，主导产业不突出，新的经济增长点不明显。城乡基础设施投入不足，城镇化水平低，教育、文化、卫生、环保等民生领域欠账较多，社会保障水平不高，城乡公共服务能力亟待提升。投资增速回落，资金、土地、环境容量等要素制约日益凸显，一些潜在风险不容忽视。政府职能转变、简政放权还不到位，体制机制障碍依然较多，少数部门和工作人员法治意识、执行能力不强，思想观念、工作作风、能力素质与经济发展新要求还不适应，“四风”问题仍然存在。对此，我们一定高度重视，认真解决。

　　二、关于2015年工作

　　2015年是全面深化改革的关键一年，是 “十二五”规划的收官之年，也是“十三五”规划谋篇布局之年。世界经济形势错综复杂、充满变数，中国经济发展进入新常态，增速换挡、结构调整、动力转换压力增大，我们冷静审视面临的形势：一是机遇叠加聚集开封，需牢牢把握。省委、省政府继续实施国家粮食核心区、中原经济区、郑州航空港经济综合实验区三大国家战略，加快推进高成长服务业大省建设。省委九届六次全会、省委经济工作会明确提出加快郑汴一体化发展，推动开封建设成为河南省新兴副中心城市，我市面临的发展环境和基础条件都处于历史上最好的时期之一。二是借力发力、顺势崛起的态势已经形成，需坚定信心。经过多年坚持不懈的奋斗，开封已经步入发展快车道，我市借自身发展之力、借政策机遇之力、借区域优势之力、借挖潜提升之力，发展速度连续4年超过全省平均水平，主要经济指标增幅继续位居全省前列，产业结构出现可喜变化，城乡面貌明显改观，发展自信显著增强，已经出现新兴的兆头，展现出光明前景。三是“双重使命”的历史定位寄予厚望，需奋发图强。开封的发展不仅事关开封500万人民的福祉，也关系到中原崛起、河南振兴、富民强省目标的实现。省委、省政府对开封发展寄予厚望，要求我们自觉担当起造福开封人民和为中原崛起作贡献的双重使命，我们必须牢牢把握发展主动权，赢得竞争中的主动、发展中的主动。四是人民群众对建设“四个开封”的美好期待，需务实苦干。建设“四个开封”事关群众福祉，是做好政府工作的不竭动力，我们只有一件一件务实事、一项一项抓落实、一步一步求突破，不断开创发展新局面，才能不辜负全市人民的信任和重托，才能做到以史为鉴、奋发图强、抢抓机遇、再创辉煌。

　　今年政府工作的总体要求是：以邓小平理论、“三个代表”重要思想、科学发展观为指导，全面贯彻党的十八大和十八届三中、四中全会精神，深入贯彻习近平总书记系列重要讲话精神，认真落实市委决策部署，主动适应经济发展新常态，坚持“好中求快”总基调，把握“五个着力点”，打好“五大攻坚战”，实现“四个新突破”，保持经济持续平稳健康发展和社会和谐稳定，加快建设法治政府、服务政府、责任政府、廉洁政府，加快建设实力开封、文化开封、美丽开封、幸福开封，加快建设河南省新兴副中心城市。

　　今年经济社会发展的主要预期目标是：在优化结构、提高质量、改善环境基础上，生产总值增长9%左右，在实际工作中努力争取更好结果；一般公共预算收入增长13%；规模以上工业增加值增长13%左右；全社会固定资产投资增长17%；社会消费品零售总额增长13%；进出口总值增长8%；实际利用外商直接投资增长7%；城镇新增就业5.5万人以上；城乡居民收入与经济同步增长；居民消费价格涨幅控制在3%左右；节能减排和环境质量改善完成省下达任务；发展方式转变取得新进展。

　　各位代表！

　　实现上述要求和目标，就必须适应“一个新常态”，就是遵循经济规律，科学研判形势，敏锐捕捉机遇，努力适应和引领经济发展新常态，努力在区域竞争中抢占先机。就必须坚持“一个总基调”，就是“好中求快”，“好”就是切实把推动发展的立足点转到提高质量和效益上来。“快”就是提速增效，确保主要经济指标增幅持续位居全省前列。就必须把握“五个着力点”，就是着力扩大投资促进消费，着力结构调整产业升级，着力深化改革扩大开放，着力科技引领创新驱动，着力保障和改善民生。就必须打好“五大攻坚战”，就是继续打好工业强市、城乡一体化示范区五年成规模、建设国际文化旅游名城、推进新型城镇化、农业产业化提升攻坚战。就必须实现“四个新突破”，就是解决经济总量小，在增加总量上实现新突破；解决产业结构不合理，在产业结构优化调整上实现新突破；解决主导产业不突出，在培育主导产业和龙头企业上实现新突破；解决新增长点不足，在培育新增长点上实现新突破。

　　今年，重点抓好以下十个方面的工作：

　　（一）做大做强工业经济。坚持工业强市不动摇，全面提高工业经济总量和质量，增强工业支撑作用。

　　壮大主导产业规模。以装备制造、化工、纺织服装、食品、木业等传统优势产业为支撑，以汽车（电动车）及零部件、电子信息、新材料、光伏、生物医药等新兴产业为突破口，加快产业结构升级步伐，壮大十大产业集群规模，力争十大产业集群主营业务收入增长15%左右，努力使主导产业更加明晰。继续提升产业集聚区“四集一转”水平，完善路网、信息、物流等基础设施配套，提升生产性服务功能，做好延链、补链和研发平台建设，力争8个产业集聚区完成固定资产投资增长20%以上。

　　培育骨干龙头企业。深入开展服务企业活动，着力帮助企业解决融资、用工、环境、土地等难题。认真抓好50家重点培育企业和50家高成长型企业发展，力争晋开集团产值超80亿元，全市主营业务收入超20亿元企业在10家以上。在向引入要增量的同时，加大向存量扩张要增量，推进企业战略重组与合作，帮助已经落户的企业做大做强。加快企业上市步伐，力争在新三板挂牌上市企业达到10家。加快工业项目建设，重点推进晋开百万吨液体肥料及60万吨复合肥、东大化工整体搬迁等127个重点工业及高新技术项目建设，确保工业项目投资突破300亿元。

　　创新驱动转型升级。优化创新生态，扶持创新文化，加快推进以科技创新为重点的全面创新，积极推动要素驱动向创新驱动转变，让创新活力竞相迸发。围绕国家重点发展的七大战略性新兴产业，谋划、包装和引进一批重点科技创新企业和项目。充分发挥政府资金对创新驱动的引导作用，实施科技创新平台建设工程，加快企业孵化平台、研发平台、资源共享平台和交流合作平台建设，推动“双50”企业建设的高水平技术研发中心。强化产学研用结合，进一步增强空分、汽车（电动车）及零部件制造等行业自主创新能力。实施科技企业孵化器三年行动计划，大力扶持中小微企业创新发展。继续实施质量强市战略，培育知名自主品牌。

　　（二）大力发展第三产业。坚持把第三产业发展作为培育新优势的战略重点，放在更加重要的位置来抓，推动产业结构优化升级。

　　大力发展文化旅游产业。发挥文化旅游资源优势，深入推进国际文化旅游名城建设。继续办好 “两节一论坛一赛事”。加快宋都古城文化产业园区建设，做好七盛角、东京极地海洋馆申报国家A级景区工作，抓好清明上河城、宋都文化商务区、城摞城新郑门遗址、朱仙镇国家生态文化旅游示范区等重点文化旅游项目建设，完成刘青霞故居改造、汴京公园及周边环境改造工程，建成中原明珠文化旅游产业园、建业东京梦华半亩园，启动城墙保护六期、地下城墙展示馆、小宋城二期等项目建设。谋划实施黄河生态旅游观光带，加快开封旅游向观光、休闲、养生、体验游转型，打造文化旅游产业新亮点。积极创建全国旅游标准化示范城市，加快建设旅游集散中心、购物中心，不断完善旅游咨询体系，全面提升景区服务质量和旅游行业信息化水平，促进旅游接待量和综合收入持续较快增长。积极融入丝绸之路文化产业带，大力推动“宋文化”走出去，叫响开封文化旅游品牌。

　　深入推进中心商务区和特色商业区建设。加快河南煤化研发中心、开封国际农业会展中心等42个亿元以上中心商务区和特色商业区重点项目建设，完成博物馆、规划馆主体工程，推进国际文化旅游博览中心、体育中心建设，开工一批商业综合体和城市综合体。加快双龙巷、珠玑巷二期、徐府街等特色商业街区建设。大力发展总部经济、智慧商务经济，培育一批主业突出、特色鲜明的商务楼宇。促进传统批发市场向有机融合电子商务、物流、会展等新型业态的现代市场转型。

　　提升服务业发展水平。坚持现代服务业与传统服务业并举，生产性服务业与生活性服务业并重，全面落实支持服务业发展的财税、价格、土地、融资等政策，推动现代物流、金融服务、电子商务等现代服务业扩量提质发展，加快建立具有多元活力的服务业发展体系。积极发展现代物流业，加快上海申华汽车文化博览园、华晨申华物流产业园、上亿商贸物流城等物流项目落地。着力发展金融服务业，推进开封国际金融中心等项目建设，积极引进各类金融机构后台服务中心。加强与知名电子商务企业合作，大力发展电子商务，全力推进中国中部电子商务港建设。扶持健康服务、教育培训、商务服务、养老及家庭服务业快速发展。合理引导市场预期，促进房地产业健康可持续发展。

　　（三）着力打造示范区核心增长极。坚持以城建出形象，以产业强支撑，把城乡一体化示范区打造成为全市经济社会发展核心增长极。

　　继续加强基础设施建设。加快启动区、铁南区、运粮河组团、水稻乡“四大片区”45个基础设施项目建设，重点推进“一湖三站两馆两中心”建设，不断完善西湖园林景观，健全郑开城际铁路宋城路站、运粮河站和郑徐客运专线开封站周边配套基础设施，抓好绿地城、红星美凯龙、北大资源城等郑开大道两侧重大项目建设，打造示范区建设新亮点。

　　着力做大新兴产业。坚持招大引强，进一步壮大汽车及零部件、农业机械、空分设备三大制造基地规模，培育电子信息、生物医药、现代服务、文化创意等新兴产业，重点抓好奇瑞微车、祥华北奔、牧宝实业等汽车及零部件项目，德豪光电、智能手机及配件等电子信息项目，方欣生物等生物医药项目，家易择配、土小宝等电子商务项目建设。

　　统筹城乡示范发展。加快水稻乡生态农业区规划实施，抓好沿黄文化旅游、生态农业等产业发展。巩固水源地周边环境综合整治成果，实施黑池、柳池水源地一级保护区内村庄搬迁。加快迁村并居步伐，推进辛堤头城市综合体项目建设，抓好马市街、演武庄、瞿家寨、野场、王府寨等29个“两改一建”项目。

　　（四）扎实推进新型城镇化。坚持“以人为本、优化布局、生态文明、传承文化、产城互动、科学有序”原则，加快构建 “两核六组团” 新型城镇化发展格局，打造现代城镇体系。

　　加快中心城区建设。编制《谋建河南省新兴副中心城市规划纲要》，优化城市格局，拓展城市功能，提升开封战略地位。深入推进郑汴一体化发展，扎实做好“五同城一共享”，推动港尉新区和郑州航空港经济综合实验区深度融合发展，把开封打造成郑汴都市区重要一翼。以老城区和城乡一体化示范区为核心，保护改造老城，开发建设新城，推动中心城区组团同步协调发展。开展“两改一建”攻坚行动，城区完成棚户区、城中村改造250万平方米，着力打造“外在古典、内在时尚、宋韵彰显”的新宋风城市风格，体现老城区改造中的商业价值和文化价值。完善老城区基础设施，启动水系一期（广济河段）改造，加快水系二期周边开发，继续实施火车站周边地区综合改造提升工程，完成华夏大道改造、迎宾路北延、内环路改造、内顺城路改造、魏都路改造、夷山大街北延等道路工程，进一步完善停车场、雨水泵站等配套设施。推进运粮河组团发展，打造融科技、教育、文化于一体的生态休闲宜居区，加快建设西部新城区。启动老工业区搬迁改造，加快祥符区规划调整，以华夏大道等道路贯通为突破口，实现东部城区与中心城区统筹协调发展。

　　抓好县城和中心镇建设。以扩大县城规模、提升县域经济实力为重点，继续加快县城建设，努力把县城打造成为吸纳农业人口转移的主阵地。提高县城规划前瞻性，加快县城升级改造步伐，三县完成房屋征收20万平方米，完善公共服务配套设施，提高县城辐射带动能力。依托各县区主导产业、特色资源，坚持规划引领、产业支撑、项目带动、城乡统筹，以新型城镇化为载体，持续做大县域经济总量，争取主要经济指标在全省排序明显前移。按照“因地制宜、科学规划、打好基础、建管并重”原则，各县选择1～2个条件优越、产业基础好、发展潜力大的中心镇，发展特色主导产业，提升城镇发展质量。坚持“一基本两牵动”，有序推进农业转移人口市民化，提高城镇化率。

　　着手打造综合交通体系。围绕“通高铁枢纽便利化、通机场枢纽便利化”，健全完善城市大交通格局，重点加快“三条高速”、“三条铁路”项目建设。推进郑徐客运专线、新开铁路改扩建开封段建设，启动郑开城际铁路延长线前期工作。完成郑民高速二期开封段主体工程，确保商登高速、机西高速开封段建成通车。力争打通连霍高速开柳路出入口。升级改造干线公路，完成省道213、218、220改造工程，提高市县间通行能力。实施公交优先战略，加强公交场站和综合枢纽站建设，再新增城市公交车150台。

　　创新城市管理服务。巩固国家卫生城市创建成果，落实好十大“开封规矩”，抓好市容环境、市政设施、园林绿化等方面的精细化管理，积极创建国家园林城市。加强“智慧开封”建设，加快建成一批成熟的智慧应用系统，提高城市数字化、动态化、实时化管理服务水平。

　　（五）统筹做好“三农”工作。按照“稳粮增收、提质增效、创新驱动”的总要求，稳定粮食生产，加快转变农业发展方式和生产方式，进一步释放农民增收、农业增效、农村发展的潜力。

　　大力发展现代农业。加快高标准粮田“百千万”工程建设，再建成高标准粮田40万亩，稳定提高粮食综合生产能力，力争全市粮食产量实现“十二连增”。强化农业科技支撑作用，积极推进3个省级农业科技园区建设，加快农业科技创新和重大技术成果转化，加强良种繁育和推广，推动主要农作物生产全程机械化。强化水利基础保障作用，加快中小河流治理、病险水闸除险和小农水重点县等水利工程项目建设，完善引黄灌溉体系，力争每县建设一处引黄调蓄工程，新增改善有效灌溉面积30万亩以上。加快推进气象现代化，增强气象防灾减灾和服务能力。

　　提升农业产业化水平。实施现代农业产业化集群培育工程，打造粮食加工、油脂加工、畜禽产品加工等八大农业产业化集群，支持农业产业化龙头企业发展壮大，重点扶持以天丰、天源、宇东等为主的粮食加工业，以龙大植物油、包公食品为主的油脂加工业，以大用、皓月、雏鹰等为主的畜禽产品加工业，以阿凡达、华誉木业等为主的林板加工业，力争现代农业产业化集群达到20个。抓好中原农产品物流园区、宏进农产品批发市场、杞县大蒜市场等30个农产品批发市场的建设运营，促进农产品流通。大力发展专业大户、家庭农场、农民专业合作社等新型经营主体。积极实施都市生态农业发展工程，力争每个县区建设一个都市生态农业示范带动项目。

　　建设美丽乡村。按照“五规合一”要求，以县域为单位，编制完善新农村建设规划，分类推进新农村综合发展、特色发展。全面推进农村人居环境整治，继续开展清洁家园·美丽乡村创建和小城镇综合整治提升工程，重点抓好“133”示范工程，打造15个示范乡镇、30个示范村、3条美丽乡村示范带。加强农村水电路等基础设施建设，再解决41.1万农村居民和在校师生饮水安全问题，新建改造一批110千伏、220千伏输变电工程，新改建农村公路555.2公里，加快农村集贸市场、垃圾中转站、污水处理点等公共服务设施建设。加快推进已开工的新型农村社区建设，促进已建成的新型农村社区建立长效管理机制，让农村成为农民安居乐业的美丽家园。

　　多渠道促进农民增收。认真落实强农惠农富农政策，完善农产品价格形成机制，保持农产品价格合理水平。拓宽农村外部增收渠道，促进农民转移就业和创业，培育新型职业农民1000人，新增农村劳动力转移就业3.5万人。大力推进农村扶贫开发，完成扶贫开发整村推进村35个，再实现3.6万农村贫困人口稳定脱贫。

　　（六）全面深化改革。严格按照中央和省委部署，积极跟进落实改革措施，确保各项改革举措落地生根，真正解决问题。

　　深化行政体制改革。全面完成市县政府机构改革、交通运输执法和食品药品监管体制改革。完善审计制度，对公共资金、国有资产、国有资源和领导干部履行经济责任情况实行审计全覆盖。推进公益性文化事业单位改革，深化文化市场综合执法改革。完成党政机关公务用车制度改革。

　　加快财税金融改革。深化预算管理制度改革，健全政府预算体系，启动中期财政规划管理，清理规范税收优惠政策以及教育、科技等7项重点支出与财政收支挂钩事项，盘活财政存量资金，全面推进预决算公开工作。扩大“营改增”范围，调整消费税征收范围、环节和税率。积极探索和推行政府与社会资本合作新模式。创新金融服务机制，破解中小微企业融资难、成本高问题。规范金融秩序，化解金融风险，促进金融业健康发展。推进县级农信社改制组建农商行。启动农村金融改革试点，探索新农村合作金融发展有效途径。

　　继续深化农村改革。深入推进农村集体产权制度改革，完成334.7万亩农村土地承包经营权确权登记颁证。规范引导农村土地经营权有序流转，发展多种形式的适度规模经营。加快房地一体的农村宅基地使用权确权颁证，完善农村宅基地有偿退出机制，建立农村产权交易市场。稳步推进农村土地制度改革。深化集体林权制度、农电体制改革。

　　与此同时，以增强活力、提高效率为中心，深化企业改革，稳妥发展混合所有制经济，推行国有企业外派监事会制度。积极推进生态环保改革。加快水电气等资源性产品价格改革。推动不动产登记制度、科技体制等改革。

　　（七）积极扩大对外开放。坚持更加积极、更宽领域、更高层次的对外开放，大力承接产业转移，增强招商实效，以开放增后劲、促发展。

　　继续招大引强。开展“招商引资攻坚年”活动，突出抓好与500强、央企、上市公司、行业领军企业的对接引进，围绕“五大攻坚战”、“十大产业集群”、“两改一建”等中心工作逐一制定专项招商方案，力争每个产业引进2～3个投资10亿元以上的大项目，在新兴产业、现代服务业、新型业态招商方面取得实质性进展。

　　创新招商方式。充分发挥企业在招商引资活动中的主体作用，大力开展精准招商、以商招商、中介招商、专题招商、集群招商。紧盯长三角、珠三角、环渤海、京津冀等要素紧缺、项目承载力趋于饱和的区域，做好驻地招商工作。建立常态化招商机制，坚持主要领导带头外出招商，着力解决招商引资忽冷忽热问题。坚持培养与引进并举，实施人才“1851”工程，为发展提供智力支撑。

　　完善项目推进机制。坚持一个项目、一名领导、一个班子、一套方案、一抓到底的“五个一”招商引资项目推进机制，努力提高合同履约率、项目开工率、资金到位率。实施市级领导分包重大项目制度，突出抓好重点项目“329”工程，全程跟踪、全程服务、全程督导，促进项目早落地、早建成、早投产、早见效，力争全年重点项目投资突破900亿元。

　　（八）有效破解发展瓶颈。创新方式方法，破解资金、土地、生态环境等瓶颈制约，保障经济社会发展。

　　解决好资金问题。深入推进与各金融机构战略合作，发挥债券、基金作用，探索融资新模式、新机制、新办法，积极推行PPP、资产证券化、金融租赁等融资方式，引导更多社会资本投入实体经济和城市建设。认真抓好企业债券发行工作。

　　解决好土地问题。落实最严格的耕地保护制度，搞好城乡建设用地增减挂钩、人地挂钩试点。注重土地挖潜，推进土地整理与置换，盘活存量土地。着力解决批而未征、征而未供、供而未用等问题，严格落实投资强度、建筑密度、容积率等硬性约束，促进土地集约、节约利用。

　　解决好生态环境问题。继续实施蓝天工程、碧水工程、乡村清洁工程、城市河流清洁行动计划。开展“重点减排项目建设和运行管理推进年”活动，切实加强减排工作。积极推进工业企业脱硫脱硝除尘、城市燃煤锅炉拆改、黄标车和老旧车淘汰、城市施工和道路扬尘治理、秸秆燃烧整治，严控污染物排放，让群众看到治霾新成效。加强饮用水源地保护。关闭一批高污染企业，继续实施老城区雨污分流，对东护城河、黄汴河上游、化肥河、药厂河实行彻底截污，完成城市污水处理设施提标改造，建成一批重点乡镇污水处理厂。全面加强节能工作，提高能源利用效率，力争城市新能源利用率在6%以上，努力创建新能源示范城市。

　　加快林业生态市建设，加强生态文明示范区创建。严厉打击环境违法行为，坚决让损害环境者付出代价，建设天蓝水净地绿的美好家园。

　　（九）进一步优化发展环境。继续简政放权，强化效能问责，确保行政效能明显提升、服务质量明显改善、发展环境明显优化。

　　继续简政放权。进一步取消和下放行政审批事项，基本取消非行政许可审批事项，大幅减少前置审批，基本编制完成权力清单、责任清单。继续强力推进“两集中两到位”，全面清理和规范中介服务事项，探索网上审批，推行模拟审批，进一步压缩审批时限、办事环节、审批手续，解决好监管“短板”和服务滞后问题。

　　提升服务质量。完善重大项目联审联批制、项目代理制、涉企收费一费制等制度。提高市民之家运行质量，落实好 “一个窗口集中办理、一站式办成、限时办结”审批模式，持之以恒开展贴心服务、主动服务、承诺服务。积极构建服务企业长效机制，综合整治企业周边环境，推行电视问政，营造亲商安商的良好外部环境。

　　强化效能问责。坚持有责必问，继续开展效能建设年工作，严肃查处机关工作人员利用职权谋取非法利益和乱收费、乱罚款、乱摊派、乱检查的“四乱”行为，着力解决推诿扯皮、吃拿卡要、效率低下等问题。

　　（十）切实保障和改善民生。继续实施十项民生工程，大力发展社会事业。

　　推动实现更高质量就业。深入实施全民技能振兴工程，完善就业创业服务体系，落实失业保险稳岗补贴政策，降低企业失业保险费率，重点做好高校毕业生、城镇困难人员、退役军人、农村转移劳动力等群体就业工作，确保新增城镇就业5.5万人，城镇登记失业率控制在4.5%以内，始终保持“零就业家庭”动态归零。发挥创业对就业的带动作用，支持民营经济和中小微企业发展，以创业促进就业、引领就业。加大治理欠薪力度。

　　完善社会保障体系。城乡居民养老保险基础养老金标准每人每月提高15元以上，企业退休人员基本养老金再提高10%。稳步推进机关事业单位养老保险制度改革。创新社会保险服务方式，加快实施社会保障“一卡通”。继续提高城乡低保、农村五保和优抚对象补助标准。实行城乡统一的独生子女父母奖励扶助制度和计划生育家庭特别扶助制度。建立健全老年福利制度。加快县级社会福利中心建设。积极发展养老、慈善、残疾人、妇女儿童、红十字会等事业。健全留守儿童、留守妇女和留守老人关爱服务体系，全面实施临时救助制度，努力让每一个身处困境者都能得到关爱。

　　抓好保障性安居工程建设。开工建设棚户区改造安置住房9203套、公共租赁住房526套，基本建成各类保障性住房7365套，完善保障性住房周边基础设施配套。进一步健全保障房建设、分配、管理、运营机制，逐步解决困难家庭住房问题。

　　办好人民满意的教育。落实好《开封市深化教育领域综合改革的若干意见》。深入实施第二期学前教育三年行动计划，扩大公办幼儿园和普惠性幼儿园覆盖面。推进义务教育均衡发展，加快中小学布局调整，三县和城乡一体化示范区、祥符区各新建1~2所初中、小学。推动普通高中内涵特色多样化发展。加强教师队伍建设。实施职教攻坚二期工程，加快职业教育园区建设，落实普惠性中等职业教育政策，从秋季起对全日制中职在校生全免学费。启动实施特殊教育提升计划，加快推进特殊教育发展。积极鼓励民间资本兴办教育。做好困难家庭学生资助工作。支持河南大学、黄河水利学院、开封大学、开封文化艺术职业学院等高校发展。

　　提高群众健康水平。切实抓好食品药品安全监管，加强餐桌污染治理，努力构建职责明确、统一权威、全覆盖无缝隙的食品药品安全监管体系，推进食品药品安全社会共治。继续深化医药卫生体制改革，优化医疗卫生资源配置，鼓励社会资本多元化办医。加快推进全科医师临床培训基地等重大卫生项目建设，加大对各级医疗卫生人才的培养。全面推进县级公立医院改革，完善公共卫生服务均等化制度，强化乡村卫生机构一体化管理。积极做好农村居民大病保险试点工作。做好重大传染病防控救治工作。强力推进新农合住院患者按病种付费。新农合人均筹资标准提高到470元，人均基本公共卫生服务经费标准从人均35元提高到40元。加快发展中医药事业。坚持计划生育基本国策，促进人口长期均衡发展。广泛开展全民健身活动，再建成一批健身场地，提高竞技体育水平，办好第九届中国郑开国际马拉松赛、市九运会及首届全民健身大会。

　　完善公共文化服务体系。谋划建设市民综合公共文化服务中心，大力支持市图书馆、美术馆、文化馆等公共文化基础设施建设，扶持民办博物馆发展，不断充实和丰富公共文化服务内容。实施文化惠民工程，精心组织“舞台艺术送农民”、政府采购公益性演出活动、农村电影放映工程，继续推进公益性文化设施免费开放。大力倡导全民阅读，丰富群众精神生活。

　　创新社会治理。树立“大平安”理念，着眼“双安”、推进“双治”、强化“双基”，加快从传统社会管理向现代社会治理转变。全面推行重大决策社会稳定风险评估机制，推动社会矛盾调处由事后处置转向事前预防。建成并有效运行县乡村三级人民调解工作平台和网络平台，落实依法逐级走访、诉访分离和积案化解制度。加强城市社区建设，提升社区综合服务水平，做好第五届居委会换届工作。继续抓好法律援助，规范落实社区矫正。健全化解各类风险的体制机制，坚决守住不发生区域性系统性风险底线。强化安全生产，以安全开封创建为主线，狠抓隐患排查，坚决遏制重特大事故发生。创新立体化社会治安防控体系，构建“一村一警”长效机制，依法打击各种违法犯罪活动，加强应急管理，确保社会和谐稳定、人民安居乐业。

　　继续加强国防动员、国防教育和国防后备力量建设，支持开封驻军、武警部队、预备役部队、民兵和人民防空建设，深入开展双拥共建，巩固军政军民团结，推动军民融合深度发展。支持人民团体发挥桥梁纽带作用。全面贯彻党的民族宗教政策，促进民族团结进步、宗教关系和谐。加强外事、侨务、港澳、对台工作。继续做好统计、史志、地震、社科研究等工作。

　　科学编制“十三五”规划。加强战略谋划，编制新型城镇体系、综合交通体系、现代产业体系、生态环境体系、创新驱动体系、公共服务体系规划，谋划好全局性的重大项目、重大工程、重点工作和重大政策措施，提高社会参与度，编制好总体规划和各专项规划，提高规划的前瞻性、科学性。

　　三、加强政府自身建设

　　以建设法治政府、服务政府、责任政府、廉洁政府为重点，加强政府自身建设，切实增强政府执行力、公信力。

　　（一）建设法治政府。贯彻落实全面推进依法治国战略，忠实履行宪法和法律赋予的职责，做到法无授权不可为、法定职责必须为，营造公平正义的法治环境。严格依法行政考核，把政府工作全面纳入法治轨道。健全依法决策机制，规范公正文明执法。加强法制宣传教育，完成“六五”普法任务。严格执行人大及其常委会的决议决定，定期向人大及其常委会汇报工作，接受专项质询，自觉接受法律监督和工作监督。支持人民政协履行职能，主动接受人民政协民主监督。认真办理人大议案、代表建议和政协提案，主动听取各民主党派、工商联、各人民团体和人民群众的意见建议。虚心接受社会监督和舆论监督。

　　（二）建设服务政府。转变政府职能，加快工作节奏，提高工作效率。牢固树立全心全意为人民服务的宗旨意识，忠实履行服务群众、改善民生职责，建设高素质公务员队伍。坚持问政于民、问需于民、问计于民，使工作决策和工作推进建立在深厚的民意基础之上。坚持人民利益至上，扎实开展“三严三实”专题教育。严格落实中央“八项规定”以及省市有关纪律要求，严控“三公”经费，坚决防止“四风”反弹回潮。

　　（三）建设责任政府。自觉担当造福开封人民和为中原崛起作贡献的双重使命，勇于负责，敢于担当，时刻保持奋发有为、昂扬向上的精神状态。大力推进政务公开、政府信息公开，办好市长专线电话，完善各类公开办事制度。强化工作责任，加强督导检查，以“钉钉子”精神抓好省委、省政府和市委重大决策部署的落实，把集体研究决定的事项坚决落到实处。坚持实干、苦干、抓落实，培育励精图治、开拓进取的创业氛围和社会风尚。

　　（四）建设廉洁政府。严明政治纪律，严守政治规矩。严格落实党风廉政建设责任制，强化“一岗双责”，完善财政资金支出、工程项目招投标、国有资产交易等各项制度，扎牢制度笼子，从源头上预防腐败。全面加强对重点领域、重点部门和重点资金的行政监察和审计监督，严查重点领域和关键岗位腐败，以反腐倡廉实效保障发展、取信于民。

　　各位代表！

　　新常态赋予新责任，新征程承载新梦想，新奋斗创造新奇迹。今日的开封，人民有期待，时代有重托，各界在关注。让我们紧密团结在以习近平同志为总书记的党中央周围，高举中国特色社会主义伟大旗帜，在省委、省政府和市委的坚强领导下，在市人大和市政协的监督支持下，凝神聚力，团结拼搏，奋发图强，为加快建设实力开封、文化开封、美丽开封、幸福开封，加快建设河南省新兴副中心城市作出新的更大的贡献！
